# Fatal Disease of Bronchial Glands

**Published:** 1853-11

**Authors:** J. F. Hammond

**Affiliations:** The U. S. Army


### *A Case of fatal disease of the Bronchial Glands.*

BY J. F. HAMMOND, M. D., OF THE U. S. ARMY.

BARRANCAS BARRACKS, FLA., 8th Aug., 1853.

DEAR DOCTOR: I have just made an interesting post-mortem examination. I was called to a negro man, about fifty years old, a few days ago, whom I found with diarrhœa of three or four days standing, and difficult respiration; pulse a little smaller than natural, and healthy as to frequency; no pain any where except in the right hypochondrium, from an old "strain;" head clear; tongue clean; no thirst; moderate appetite; chest resonant throughout to percussion; the bronchial respiration loud; the vesicular sound nearly natural; occasionally slight cough; sounds of the heart a little obscured; a little dull pain on pressure upon the right hypochondrium. A blister plaster was applied over the pain. A bath, and castor oil followed by pills of blue mass, ipecac. and plumbi. acet, combined, relieved him of the diarrhœa in forty-eight hours. I left him at that time as convalescent. And, as I found him at my last visit breathing quietly, I concluded he had been shamming as to his respiration.

Twenty-four hours afterwards I was called to him by his owner. The difficuly of respiration was greatly increased—in every other respect he was apparently *well*, except that there was some tenderness on strong pressure over the vertebræ, from the middle of the column downwards. A blister plaster was applied over the tender part of the spine; and a Medical Officer of the Navy was called in consultation. It was determined that the upper surface of the liver was possibly inflamed, involving the diaphragm· Calomel and opium, in combination, were given him every four hours. At the end of seven hours he was dead.

The autopsy was made an hour and a half after death. The viscera of the abdomen were perfectly healthy, except some injection of the duodenum. In the thorax, the heart was healthy, the right lung was suspended by a net of old adhesions like a cocoon in its mesh; the left lung was collapsed, but there were old adhesions in many places; both were covered by a heavy coating of melanotic deposite, which in some places, most posteriorly, was spread uniformly under the surface—it seemed to form a part of the tissue as the pigmentum nigrum does of the skin; during the intervals of the deposite the pink hue was a shade deeper than natural; the lungs were crepitant and pervious to air throughout; they floated perfectly on water; they were *very* little conjested. In removing the lungs, several of the bronchial glands were cut through—they were the size of large almonds, black throughout; several of them had suppurated, and poured out, when cut, a deep bluish-black pus. Their pressure on the bronchial tubes had, doubtless, caused the death.

